# Composition and genomic organization of arthropod Hox clusters

**DOI:** 10.1186/s13227-016-0048-4

**Published:** 2016-05-10

**Authors:** Ryan M. Pace, Miodrag Grbić, Lisa M. Nagy

**Affiliations:** Department of Molecular and Cellular Biology, University of Arizona, Tucson, AZ 85721 USA; Department of Biology, University of Western Ontario, London, ON N6A 5B7 Canada; Universidad de la Rioja, 26006 Logroño, Spain; Division of Maternal-Fetal Medicine, Department of Obstetrics and Gynecology, Baylor College of Medicine, Houston, TX 77030 USA

**Keywords:** Hox, Evolution, Development, Arthropod, Chelicerate, *Tetranychus*, *Ixodes*, *Daphnia*, Segmentation

## Abstract

**Background:**

The ancestral arthropod is believed to have had a clustered arrangement of ten Hox genes. Within arthropods, Hox gene mutations result in transformation of segment identities. Despite the fact that variation in segment number/character was common in the diversification of arthropods, few examples of Hox gene gains/losses have been correlated with morphological evolution. Furthermore, a full appreciation of the variation in the genomic arrangement of Hox genes in extant arthropods has not been recognized, as genome sequences from each major arthropod clade have not been reported until recently. Initial genomic analysis of the chelicerate *Tetranychusurticae* suggested that loss of Hox genes and Hox gene clustering might be more common than previously assumed. To further characterize the genomic evolution of arthropod Hox genes, we compared the genomic arrangement and general characteristics of Hox genes from representative taxa from each arthropod subphylum.

**Results:**

In agreement with others, we find arthropods generally contain ten Hox genes arranged in a common orientation in the genome, with an increasing number of sampled species missing either *Hox3* or *abdominal*-*A* orthologs. The genomic clustering of Hox genes in species we surveyed varies significantly, ranging from 0.3 to 13.6 Mb. In all species sampled, arthropod Hox genes are dispersed in the genome relative to the vertebrate *Mus musculus.* Differences in Hox cluster size arise from variation in the number of intervening genes, intergenic spacing, and the size of introns and UTRs. In the arthropods surveyed, Hox gene duplications are rare and four microRNAs are, in general, conserved in similar genomic positions relative to the Hox genes.

**Conclusions:**

The tightly clustered Hox complexes found in the vertebrates are not evident within arthropods, and differential patterns of Hox gene dispersion are found throughout the arthropods. The comparative genomic data continue to support an ancestral arthropod Hox cluster of ten genes with a shared orientation, with four Hox gene-associated miRNAs, although the degree of dispersion between genes in an ancestral cluster remains uncertain. *Hox3* and *abdominal*-*A* orthologs have been lost in multiple, independent lineages, and current data support a model in which inversions of the *Abdominal*-*B* locus that result in the loss of *abdominal*-*A* correlate with reduced trunk segmentation.

**Electronic supplementary material:**

The online version of this article (doi:10.1186/s13227-016-0048-4) contains supplementary material, which is available to authorized users.

## Background

The Hox genes are a highly conserved set of homeodomain transcription factors that function in fundamental developmental processes in metazoans. This conservation also extends to their genomic arrangement [1–3]. They are typically found clustered in the genome, in the same transcriptional orientation, with their anterior–posterior (A/P) domains of expression and function mirroring their genomic position, commonly referred to as spatial collinearity [4–6], although in rare occurrences Hox genes are not clustered and are spread throughout the genome [7–9]. Among metazoans, Hox genes have been intensely studied in arthropods and it is hypothesized that a genomic cluster of ten genes (*labial*, *proboscipedia*, *Hox3*, *Deformed*, *Sex combs reduced*, *fushi tarazu*, *Antennapedia*, *Ultrabithorax*, *abdominal*-*A*, and *Abdominal*-*B*) was present ancestrally within the clade [1], a hypothesis further supported by data from a member of the Onychophora, the sister clade to arthropods, that has a full complement of ten Hox genes [10].

**Fig. 1:**
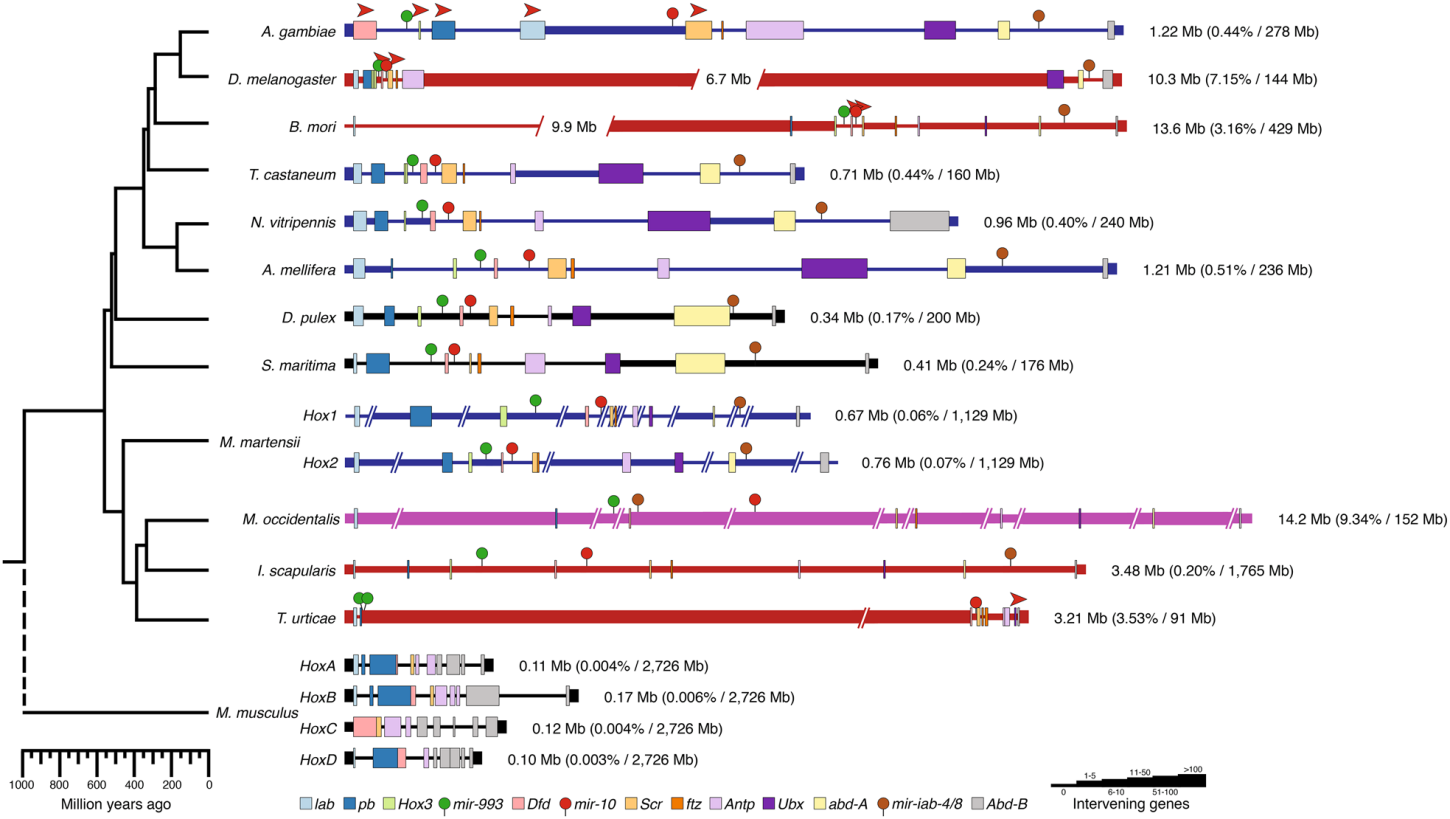
Overall size and genomic organization of arthropod Hox genes varies. On the *left* is a representative phylogenetic tree depicting relationships among the arthropod taxa used in the comparative analysis, as depicted in [32, 100–102]. *Mus musculus* is used as an out-group. *Colored boxes* represent Hox genes (and mice homologs according to [103]) and miRNAs, with numbers to the *right* of the *black line* indicating approximate size of the genomic region displayed for individual taxa. All Hox genes are depicted in the same transcriptional orientation, except where indicated with a *red arrowhead*. Data on inversions within the *Anopheles* Hox cluster are conflicting, as published data only show a single microinversion of *ftz* [13, 14, 21]; however, the most recent genome assembly shows a large inversion from *labial* to *Deformed* as depicted here. *Broken lines* indicate large genomic spans in *Bombyx* (12 Mb from *lab* to *pb*, with 9.9 Mb removed here for ease of view), *Drosophila* (9.7 Mb from *Antp* to *Ubx*, with 6.7 Mb removed here for ease of view), and *Tetranychus* (2.9 Mb from *pb* to *Dfd*). The *Anopheles*, *Tribolium*, *Nasonia*, *Apis*, and *Mesobuthus* Hox gene clusters are depicted at 1/2 scale (denoted by the *horizontal blue line*), the *Drosophila*, *Bombyx,* *Ixodes*, and *Tetranychus* Hox gene clusters at 1/6 scale (denoted by the *horizontal red line*), and the *Metaseiulus* Hox gene cluster at 1/20 scale (denoted by the *horizontal purple line*). The number of intervening protein coding genes between Hox genes is indicated by *horizontal line thickness*. *Numbers* to the *right* indicate the respective length of the Hox clusters in the genome in megabase pairs (Mb), as calculated from the transcriptional start of the most 3′ Hox gene to the transcriptional stop of the most 5′ Hox gene, and the proportion of the genome that contains the Hox cluster is indicated as a percentage along with the genome size in parentheses, respectively

While gene-based surveys in phylogenetically diverse arthropods support a general conservation of Hox genes in extant arthropods [11, 12], comparatively little is known about the conservation of the genomic arrangement of Hox genes (i.e., genomic clustering) throughout the phylum as the majority of data comes from insects. Hox genes in insects are generally positioned relatively close to each other on the same chromosome in the same transcriptional orientation, but have different amounts of intervening genes and intergenic space [13–16] (Fig. 1). However, there are several exceptions to this. For example, the *Drosophila melanogaster* Hox genes are split between the *Antennapedia* and *Ultrabithorax* complexes, separated by a large gap (~9.7 Mb) [17, 18] (Fig. 1). Only one other non-Drosophilid, the silk moth *Bombyxmori*, is known to contain a split genomic arrangement of Hox genes, where a large genic gap (~12 Mb) exists between *labial* and the rest of the Hox cluster [19, 20] (Fig. 1). Additionally, inversions that disrupt the transcriptional orientation of Hox genes are found in several taxa, including *Drosophila* and *Anopheles gambiae* [13, 14, 17, 18, 21, 22] (Fig. 1). These fragmented and inverted organizations appear unusual among insects.

There have been few descriptions of the genomic organization of arthropod Hox genes outside of insects. Two of the better characterized sets of Hox genes from genomic assemblies in non-insects include the myriapod *Strigamia maritima* [23] and the chelicerate *Tetranychus urticae* [24]. The *Strigamia* genome contains nine of the ten canonical arthropod Hox genes grouped together (missing *Hox3*), in the same transcriptional orientation [23] (Fig. 1). In *Tetranychus*, there is a large gap between *proboscipedia* and *Deformed*, duplications of *fushi tarazu* and *Antennapedia*, losses of *Hox3* and *abdominal*-*A*, and an inversion of *Abdominal*-*B* [24] (Fig. 1). These data from non-insect species suggest there is more variation in the genomic organization of arthropod Hox genes than previously appreciated. It is perhaps unsurprising then that an incomplete set of the canonical ten arthropod Hox genes is often found when performing degenerate PCR surveys [11, 25].

While the general paradigm is that most arthropods contain a similar set of ten Hox genes, there are examples where Hox gene loss or duplication (and divergence) has occurred [11, 25]. The best studied of the examples of gene loss are the *fushi tarazu* (*ftz*) and *Hox3* genes [26–31]. In the case of the *ftz* gene, loss of homeotic function, without a loss of the gene itself, appears to have been relatively common and there is currently no evidence that its loss of homeotic function had any phenotypic consequence [32]. However, here we report that loss of *abd*-*A* in *Tetranychus* [24], the oribatid mite *Archegozetes longisetosus* [11], a pycnogonid [33], and three species of barnacle [34–36]—all of which have very reduced trunk segmentation—suggest some Hox gene losses correlate with discrete morphological change along the A/P body axis.

**Fig. 2:**
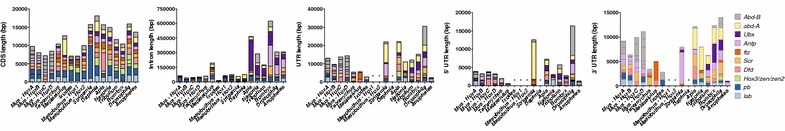
Comparison of the relative sizes of the coding, intronic, and untranslated regions of arthropod and vertebrate Hox genes. The relative sizes of amino acid coding sequence (CDS), introns, and total and 5′ and 3′ untranslated regions (UTRs) are shown for twelve arthropods surveyed and the vertebrate *Mus musculus*. Individual Hox genes are represented as a stack, in their respective genomic location of the Hox cluster, and labeled by color. The overall coding sequence length among arthropods ranges from 7.1 kb in *Ixodes* to 18.1 kb in *Daphnia*. The overall intron length among arthropods ranges from 14.5 kb in *Ixodes* to 624.6 kb in *Bombyx*. The overall UTR length among arthropods ranges from 2.3 kb in *Anopheles* to 30.5 kb in *Drosophila*. *Asterisks* indicate missing or incomplete data

In contrast to the uncommon association of a loss of a Hox gene with morphological variation, there are numerous examples where morphological diversification along the A/P body axis is achieved through changes in the regulation of the Hox genes, leading to variation in the A/P expression boundaries of the Hox genes [37, 38] (see [12] for review), as well as to changes in their downstream targets [39, 40] (see [41] for review). The intergenic regions between Hox genes are thus important sites of regulation [42] and also include microRNAs (miRNAs), small non-coding RNAs, known to play essential roles in Hox gene regulation [43]. While there is no consensus on the number of conserved arthropod Hox gene-associated miRNAs, more than twenty are functionally annotated in *Drosophila melanogaster* between the Hox genes *labial* and *Abdominal*-*B* [44], with four of these miRNAs—*miR*-*993*, *miR*-*10*, *miR*-*iab*-*4*, and *miR*-*iab*-*8*—found in conserved positions within arthropod genomes [45–51] (Fig. 2). The last two, *miR*-*iab*-*4* and *miR*-*iab*-*8*, reside at the same locus and produces sense (*miR*-*iab*-*4*) and antisense (*miR*-*iab*-*8*) transcripts [52, 53]. However, these views of arthropod Hox gene regulation are mainly derived from insects, leaving relatively little known about the extent of miRNA conservation and divergence throughout the Arthropoda.

With representative sequenced genomes from each major arthropod clade now available, we can examine the variation in the genomic organization and evolution of arthropod Hox gene clusters within a greater phylogenetic context, including the degree to which chromosomal arrangement, transcriptional orientation, and regulatory elements such as miRNAs are conserved. Using a comparative genomic approach, we examined how the spatial organization of Hox genes has changed during arthropod evolution. To this end, we performed a comparative analysis of the genomic structure of Hox genes that have been previously characterized in six insects, the centipede *Strigamia maritima* (Myriapoda) [23], several chelicerates including the scorpion *Mesobuthus martensii*, the spider mite *Tetranychus*, the predatory mite *Metaseiulus occidentalis*, and the vertebrate *Mus musculus*. In addition, we included in our comparative analysis the water flea *Daphnia pulex* (Crustacea) [54] and the deer tick *Ixodes scapularis* (Chelicerata) [55], arthropods that have yet to have the genomic organization of their Hox genes well characterized.

## Methods

### Hox genes and genomic sequence collection

#### Species choice

We focused our analysis on non-insect Hox clusters for which less information on Hox cluster organization has been published. Reliable analysis of genomic organization of a Hox cluster requires sufficiently long contigs and confidence in the accuracy of the assembly. Consequently, we limited our analysis to those genomes with sufficient quality coverage and assembly (at least 7X coverage with Sanger sequencing, or 15X coverage with 454 pyrosequencing) and for which the data were publically available. At present, six of the published genomes of non-insect arthropods adhere to that standard and are included in our analysis. We note, however, that for none of these species is there a chromosome linkage map; so validating contig linkage relationships is not possible. We also included representatives of the major clades of insects for which a completed sequence was available and focused on the best-quality genome assemblies (6 species).

*Databases used*: *Tetranychus urticae* data were collected from the Online Resource for Community Annotation of Eukaryotes (OrcAE, http://bioinformatics.psb.ugent.be/orcae/) [56]. *Daphnia pulex* data were collected from the JGI Daphnia pulex v1.0 genome database (http://genome.jgi-psf.org) [54]. *Strigamia maritima* data were collected from the EnsemblGenomes database (http://metazoa.ensembl.org/) [23]. *Ixodes scapularis* and *Anopheles gambiae* data were collected from the VectorBase IscaW1.4 and AgamP4 databases, respectively (https://www.vectorbase.org) [55, 57, 58]. *Bombyx mori* data were collected from the Silkworm Genome Database (http://www.silkdb.org/silkdb) [59] and from the work of [19, 20]. *Apis mellifera* data were collected from the Hymenoptera Genome Database (http://hymenopteragenome.org/) [60]. *Drosophila melanogaster*, *Nasonia vitripennis*, *Tribolium castaneum*, *Metaseiulus occidentalis*, *Mesobuthus martensii*, and *Mus musculus* data were collected from NCBI. Gene accession identification numbers are included in Additional file 1: Table S1.

### Annotation of conserved arthropod Hox gene-associated miRNAs

In identifying conserved miRNAs, we undertook a sequence homology-based approach that was consistent with previous studies [45, 61–64]. Briefly, Hox gene-associated miRNAs were first curated from miRBase [44] by searching the genomic positions of the Hox genes for *Bombyx*, *Drosophila*, *Anopheles*, *Apis*, *Nasonia*, *Tribolium*, *Daphnia*, and *Ixodes*. *Drosophila* Hox gene-associated miRNA stem-loop sequences were then downloaded from miRBase and used in BLAST analyses to identify conserved Hox gene-associated miRNAs in *Tetranychus*, *Mesobuthus*, *Metaseiulus*, and *Strigamia*. Identification of putative precursor miRNA sequences was based on a BLAST hit with an alignment length greater than or equal to 20 nucleotides and greater than or equal to 80 percent identity (Additional file 2: Table S4). BLAST hits were then analyzed for predicted secondary structure using minimum free energy (MFE) with RNAfold [65] and miRAlign [61]. Only sequences with a predicted stem-loop structure with a MFE less than or equal to -20 kcal/mol and that contained the mature sequence on the stem were considered putative miRNAs (Additional file 2: Tables S4, Additional file 3: Table S5). Predicted precursor miRNA sequences were aligned to other arthropod miRNA sequences using MUSCLE [66], trimmed, and subsequently used in generating a maximum likelihood phylogenetic tree [67] to support orthology as previously described [68], using the HKY85 substitution model (Additional file 4: Figure S1; Additional file 5). Accession numbers or genomic locations of miRNAs are included in Additional file 1: Table S1.

## Results

### Comparison of the genomic arrangement of arthropod Hox genes

In Fig. 1, we compare key features of the genomic organization of the insect, non-insect and mouse Hox genes, including relative position on the chromosome, intergenic spacing, number of intervening genes, transcriptional orientation, and the position of Hox gene-associated miRNAs.

*Overall size of the Hox cluster:* None of the arthropod Hox clusters analyzed show the tightly linked genomic organization as seen in the vertebrate *Mus musculus,* in which Hox clusters range from 0.10 to 0.17 Mb in size (Fig. 1 and Additional file 6: Table S2). *Daphnia* contains the tightest linked arrangement of Hox genes observed in any arthropod with a sequenced genome to date (0.34 Mb; Fig. 1; Additional file 6: Table S2). The ten *Daphnia* Hox genes are located on a single genomic scaffold (scaffold 7), which measures 2.3 Mb in length. The *Ixodes* Hox genes are also located on a single genomic scaffold (DS891538), which measures 3.9 Mb in length, but the *Ixodes* Hox genes span ten times the genomic distance of the *Daphnia* Hox genes (3.48 Mb; Additional file 6: Table S2). The increase in the spatial arrangement of the *Ixodes* Hox cluster is correlated with its particularly large genome (1.7 Gb) [57] relative to other arthropods with sequenced genomes, which range from 91 Mb in *Tetranychus* [24] to 1.3 Gb in *Aedes aegypti* [69]. However, the *Ixodes* Hox cluster occupies a similar percentage of the genome (0.20 %) as other arthropod Hox clusters that lack large genomic gaps (0.3 ± 0.2 %, mean ± standard deviation).

#### Splits in the Hox cluster

The *Drosophila* Hox cluster is the best-known example of a split Hox cluster, in which the *Antennapedia* complex is separated from the genes in the *Bithorax* complex by 9.6 Mb. A similarly large split is evident in the *Bombyx* Hox cluster (12 Mb), but separates *labial* from the rest of the Hox genes [17–20]. We also observed a split in the Hox gene organization of *Tetranychus* [24]. The *Tetranychus* Hox genes are located on two genomic scaffolds: A 2.7-Mb scaffold (genomic scaffold 11) contains orthologs of *proboscipedia* (*Tu*-*pb*) and *labial* (*Tu*-*lab*) in a shared 5′ to 3′ orientation, and a 1.6-Mb scaffold (genomic scaffold 20) contains the remaining Hox genes *Deformed* (*Tu*-*Dfd*), *Sex combs reduced* (*Tu*-*Scr*), *fushi tarazu* (paralogs 20g02520 and 20g02530, respectively), *Antennapedia* (paralogs 20g02430 and 20g02440, respectively), *Ultrabithorax* (*Tu*-*Ubx*), and *Abdominal*-*B* (*Tu*-*Abd*-*B*) (Fig. 1). At this time it is not possible to further align the genomic scaffolds into a contiguous sequence due to the holocentric nature of the three chromosomes of *Tetranychus* [24]. Presuming the scaffolds are contiguous in the genome, the gap between the *Tu*-*pb* locus and the *Tu*-*Dfd* locus is a minimum distance of ~2.9 Mb (2.47 Mb from the stop codon in *pb* to the 3′ end of scaffold 11 and 0.47 Mb from start codon in *Tu*-*Dfd* to the 5′ end of scaffold 20) and contains more than 100 predicted and manually annotated genes (Fig. 1). Thus, the *Tetranychus* Hox cluster split is unique in both where it is located (between *pb* and *Dfd*) and the number of genes interspersed in the region (Fig. 1). However, when the region between *Tu*-*pb* and *Tu*-*Dfd* is excluded, the length from the most 5′ to the most 3′ Hox gene in *Tetranychus* is reduced compared to most insects. For example, the *Scr* and *ftz* genes are a mere 1.1 kb apart (Fig. 1 and Additional file 6: Table S2).

We note that for species where Hox genes are found on separate genomic scaffolds (*Tetranychus*, *Metaseiulus*, and *Mesobuthus*), we have arranged the genomic scaffolds to reflect the expected genomic organization of Hox genes (Fig. 1). These data should be interpreted to represent the *minimum* genomic arrangement, and chromosome linkage mapping is needed to confirm this arrangement. It is possible that there are large genomic regions between Hox gene-containing scaffolds in both the scorpion and predatory mite, and an even larger than depicted gap between the *Tetranychuspb* and *Dfd* genes, but until the scaffolds can be further linked it is not possible to ascertain.

### Transcriptional orientation

With very few exceptions, the Hox genes share the same transcriptional orientation on their respective chromosomes. In our sample, inversions are limited to the derived insects, *Drosophila*, *Bombyx*, and *Anopheles* [17–20]; and the *Abd*-*B* gene in *Tetranychus* [24] (Fig. 1). The inversion of *Tu*-*Abd*-*B* is also found in two other tetranychid mites (*T. lintearius and T. evansi*, separated by 0.8 and 3 MYA, respectively; data not shown). This inversion is consistent with a model in which the loss of *abdominal*-*A* resulted from a chromosomal inversion that spanned both the *abd*-*A* and *Abd*-*B* loci at the base of this lineage.

### Lineage-specific Hox gene duplications

As previously reported [24], several instances of Hox gene duplications were identified in *Tetranychus*. *Tetranychus* harbors two copies of both *fushi tarazu* and *Antennapedia* as tandem duplications, not present in other arthropods (Fig. 1). Needleman–Wunsch global alignment of the coding sequence shows the *Tu*-*ftz1 and Tu*-*ftz2* and *Tu*-*Antp1* and *Tu*-*Antp2* orthologs share 74 and 55 percent identity, respectively, and at the amino acid level 67 and 33 percent identity, respectively (Additional file 7). Phylogenetic analysis of representative arthropod *ftz* and *Antp* sequences supports a hypothesis that the *Tetranychusftz* and *Antp* paralogs emerged via lineage-specific duplications (Additional file 8: Figure S2). The percent identity for the *Tetranychus* duplicate *ftz* and *Antp* orthologs is consistent with another well-known tandem gene duplication located in an arthropod Hox cluster; e.g., *Triboliumzerknüllt* and *zerknüllt*-*2* share 54 % nucleotide identity and 37 % protein identity, respectively. Alignment of the *Tetranychusftz* and *Antp* amino acid sequences suggests either partial duplications of the genes including the homeodomain-containing region, or deletions occurred post-duplication, upstream of the homeodomain in *Antp2440* and *ftz2530* as the putative coding sequence for both the *ftz* and *Antp* duplicates is substantially smaller (Additional file 8: Figure S2). Additionally, comparison of RNAseq profiles across four developmental stages (RNASeq from embryonic, larval, nymphal, and adult) reveal both *Antp2440* and *ftz2530* have markedly lower levels of expression than their respective paralogs (Additional file 9: Table S6). The scorpion genome revealed the presence of two complete Hox clusters (Fig. 1) [70]. Surveys of the remaining non-insect genomes sampled revealed no apparent Hox gene duplications.

### Individual Hox gene characteristics

To further explore variation in Hox gene genomic complexes, we characterized the structure of the *Tetranychus*, *Daphnia*, and *Ixodes* Hox genes and compared them to *Anopheles*, *Drosophila*, *Bombyx*, *Tribolium*, *Nasonia*, *Apis*, *Strigamia*, *Metaseiulus*, *Mesobuthus*, and *Mus musculus* (Fig. 2). In general, we find chelicerates to have reduced amounts of coding sequence, intronic regions, and untranslated regions (Fig. 2). There is little variation in the coding sequence of myriapod, crustacean, and insect Hox genes. Compared to insects, chelicerates, *Strigamia*, and *Daphnia* contain reduced Hox gene intronic regions (Fig. 2). Similarly, where there are available data on the 5′ and 3′ untranslated regions (UTRs), the UTRs of chelicerate and *Daphnia* Hox genes are reduced compared to insects. For example, the average total intron length and intron number for Hox genes in *Tetranychus* are 5.32 kb and 2.0 introns, in *Ixodes* are 1.45 kb and 0.9 introns, and in *Daphnia* are 7.61 kb and 4.0 introns (Fig. 2 and Additional file 10: Table S3). In contrast, the average total intron length and intron number for Hox genes in *Drosophila* are larger at 25.66 kb and 3.8 introns (Fig. 2 and Additional file 10: Table S3). Similarly, the average length of untranslated regions of *Tetranychus* and *Ixodes* Hox genes (0.49 and 0.31 kb, respectively) is reduced compared to *Drosophila* (2.54 kb) and other arthropods (Fig. 2 and Additional file 10: Table S3). The decrease in untranslated regions and introns of individual Hox genes effectively reduces the size of the overall transcription units in *Tetranychus* and *Ixodes* (Fig. 2 and Additional file 6: Table S3). In *Tetranychus*, these features are not specific to the Hox cluster as a reduction of intergenic regions, introns, and untranslated regions is general features of the highly compact *Tetranychus* genome [24]. Nonetheless, the reduction in putative regulatory DNA associated with *Tetranychus* Hox genes has implications for their regulation during development.

### Hox gene-associated miRNAs

We identified multiple sequences with homology to the four conserved miRNAs (i.e., *mir*-*993*, *mir*-*10*, *mir*-*iab*-*4*, and *mir*-*iab*-*8*) in *Tetranychus* (3/4), *Mesobuthus* (4/4), *Metaseiulus* (4/4), and *Strigamia* (4/4) (Fig. 1, Additional file 4: Figure S1; Additional file 2: Table S4, Additional file 3: Table S5). There are two sequences in *Tetranychus* separated by less than 500 bp that contain homology to *mir*-*993* located near *pb* (Fig. 1). As *mir*-*993* is typically found conserved between *Hox3* and *Dfd*, the position of the *Tetranychusmir*-*993* homologs adjacent to *Tu*-*pb* provides additional support for the complete loss of *Hox3* from the genome. In *Tetranychusmir*-*10* is located upstream of *Tu*-*Dfd*, differing from its expected conserved location between *Dfd* and *Scr* (Fig. 1). We were unable to locate *mir*-*iab*-*4*/*mir*-*iab*-*8* in *Tetranychus*, which typically overlap each other in the region between *abd*-*A* and *Abd*-*B* (Fig. 1). The absence of *mir*-*iab*-*4*/*mir*-*iab*-*8* from *Tetranychus* further validates the loss of *abd*-*A* from the *Tetranychus* genome. All four *Mesobuthus* and *Strigamia* miRNAs were found in the expected genomic positions: *mir*-*10* is located between *Dfd* and *Scr*, *mir*-*iab*-*4*/*mir*-*iab*-*8* is located between *abd*-*A* and *Abd*-*B*. In *Mesobuthus*, *mir*-*993* lies between *Hox3* and *Dfd*, whereas in *Strigamia*, despite the loss of *Sm*-*Hox3* from its conserved position between *pb* and *Dfd*, *mir*-*993* is located between *pb* and *Dfd* (Fig. 1). *Metaseiulus* contains a unique arrangement of Hox gene-associated miRNAs; both *mir*-*993* and *mir*-*10* are found on non-Hox gene-containing scaffolds, whereas *mir*-*iab*-*4*/*mir*-*iab*-*8* is located on the *Dfd*-containing scaffold (Fig. 1).

## Discussion

### Changes in the genomic organization of arthropod Hox genes

To understand the conservation of Hox gene clustering throughout the arthropod phylum, we performed an arthropod-wide comparison of the genomic structure of Hox genes from representative arthropod genome sequences. We analyzed and compared the genomic organization of Hox genes from several insects, a crustacean, a myriapod, four chelicerates, and the vertebrate *Mus musculus*. Our data suggest the constraints maintaining Hox genes in a tightly clustered genomic complex have been lost during arthropod evolution (Fig. 1), although the sample size remains relatively small (e.g., there are an estimated 2–10 million arthropod species [71]). Excluding arthropods with Hox genes organized into distinct genomic complexes (e.g., *Drosophila*, *Bombyx*, and *Tetranychus*), the variation in clustering of Hox genes is mainly attributed to the amount of intervening space between Hox genes, not to a lack of Hox gene coding sequence, intron, and UTR lengths. For example, intervening space accounts for 0.25 Mb (72 %) of the *Daphnia* Hox gene cluster. In contrast, intervening space accounts for 3.45 Mb (99 %) of the Hox gene cluster in *Ixodes*. There does not appear to be a phylogenetic trend for proportion of intervening sequence. Furthermore, large gaps in the genomic arrangement of Hox genes have independently occurred in insects and chelicerates with sequenced genomes to date (Fig. 1). These large breaks do not appear at consistent locations within the cluster (Fig. 1). For example, the genomic split within the *Tetranychus* Hox genes represents a novel arrangement of Hox genes compared to those in dipteran and lepidopteran genomes.

The predatory mite *Metaseiulus occidentalis* was reported to have a disintegrated arrangement of Hox genes in the genome—each Hox gene is localized to a separate genomic scaffold, that if contiguous on a single chromosome would span approximately 12.1–14.2 Mb [72] (Fig. 1). While *Metaseiulus*, *Tetranychus*, and *Ixodes* are all are members of the chelicerate subclass Acari, *Metaseiulus* is more closely related to *Ixodes* (both are members of the Parasitiformes superorder, and *Tetranychus* is a member of the Acariformes superorder). The *Ixodes* Hox genes were uncovered on a single large scaffold, but the Hox genes span 3.5 Mb of the genome and none of the genes are tightly clustered in this region (Fig. 1). Thus, these data suggest that compared to other arthropods, there may have been fewer constraints on mites and ticks to maintain the paradigmatic clustered arrangement of Hox genes.

While the mechanisms that constrain Hox genes into genomic clusters during evolution are not well understood, they are believed to be tied to their temporal collinearity in vertebrates [73]. The evolution of rapid development and simultaneous appearance of segments in flies (22 h [74]) has been proposed as a key factor in the loss of temporal collinearity and thus disintegration of Hox gene clusters [9]. Although it not known whether *Tetranychus* Hox genes are temporally collinear, *Tetranychus* embryonic development is also relatively short (~39 h [75]). The development of *Bombyxmori*, however, contradicts this model. While *Bombyx* have a split arrangement of Hox genes, *Bombyx* embryos take 10 days to complete embryogenesis [76]. Interestingly, in several insects and in *Strigamia*, expression of the posterior class Hox genes is temporally collinear [77–80]. Taken together, these data, or more so, lack of data, reveal that the temporal expression of Hox genes in arthropods is under-appreciated and under-studied. It is possible that shifts in the temporal collinearity of Hox gene expression, like anteroposterior shifts in expression domains, should also be considered as a driver of morphological change.

### Conservation of miRNAs in arthropod Hox clusters

Four miRNAs located in close proximity to Hox genes are found conserved throughout Arthropoda—*mir*-*993*, *mir*-*10*, *mir*-*iab*-*4*, and *mir*-*iab*-*8*. To date there has only been one arthropod identified that contains duplicated copies. *Bombyx* has two copies of *mir*-*993*—*mir*-*993a* is present in the Hox cluster in the expected position between *pb*/*Hox3* and *Dfd*, whereas *mir*-*993b* is located on a genomic scaffold separate from the scaffolds that contain the Hox genes [51]. Similarly, *Tetranychus* contains two copies of *mir*-*993*, although both are in the expected position downstream of *pb* (Fig. 1). However, the *mir*-*993* duplications in both *Bombyx* and *Tetranychus* likely represent lineage-specific duplications, as they do not cluster together in a phylogenetic analysis (Additional file 4: Figure S1). We also found *Tetranychus mir*-*10* has translocated from a conserved position between *Dfd* and *Scr* to upstream of *Tu*-*Dfd* (Fig. 1). *Strigamia* contains a single copy of the four conserved Hox gene-associated miRNAs, and we did not find any evidence for translocation (Fig. 1). We speculate that the losses of *mir*-*iab*-*4* and *mir*-*iab*-*8* from the *Tetranychus* genome reflect the loss of the nearby Hox gene *abd*-*A*. Notably, in *Drosophila*, *mir*-*iab*-*4* appears to be transcriptionally co-regulated with *abd*-*A* and *mir*-*iab*-*8* plays a regulatory role in the expression of *abd*-*A* and *Ubx* and maintaining posterior segment identities during early patterning [53, 81]. In *Tetranychus*, the loss of the region containing *abd*-*A*, *mir*-*iab*-*4*, and *mir*-*iab*-*8* may have contributed to the reduced posterior patterning observed (described in further detail below). However, an examination of the expression and function of *mir*-*iab*-*4* and *mir*-*iab*-*8* in more phylogenetically basal arthropods, such as chelicerates, remains to be determined.

### Hox gene duplications

Despite all extant arthropods containing the same basic set of Hox genes [1, 12], there are an increasing number of examples of lineage-specific Hox gene duplications. Duplications of Hox genes have been previously observed via PCR surveys in several non-insect arthropods, e.g., *Scr*, *Dfd*, and *Ubx*, in the spider *Cupiennius salei* [82] and *Dfd* in the centipede *Pachymerium ferrugineum* [83]. Additionally, PCR and genomic surveys in horseshoe crabs suggest the entire Hox gene complex has undergone several duplications, potentially via whole genome duplication [84, 85], and similar large-scale duplications of Hox genes have been observed in scorpions [70, 86] (Fig. 1). Where available, analysis of gene expression data reveals the duplicated genes to have overlapping, but distinct spatial domains [82, 86]. We also observed Hox gene duplications in *Tetranychus* for *ftz* and *Antp*. Whether the *Tetranychus* duplicated Hox genes also contain overlapping, but distinct spatial domains of expression awaits examination. However, both *Tetranychus* duplicated Hox genes are truncated in length suggesting potential pseudogenization of the loci. Consistent with a model of pseudogenization, both *Tetranychus* duplicated Hox genes have lower RNASeq expression profiles compared to their respective paralogs (Additional file 9: Table S6), typical of a gene duplication event in which one duplicate is free to lose function retained by the other duplicate [87–89].

### Loss of *abd*-*A* and evolution of arthropod posterior segmentation

**Fig. 3:**
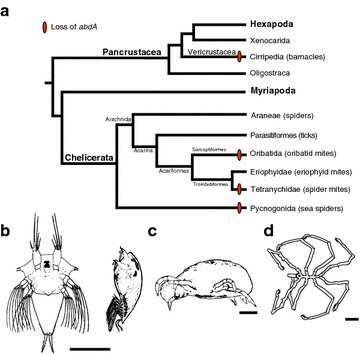
Reconstruction of the pattern of *abdominal*-*A* loss within the major clades of arthropods and their correlation with a reduction in posterior segmentation. **a** Phylogenetic relationships of arthropods based on previous data [101, 104–106]. Arthropod taxa from at least two subphyla that have been identified as missing *abd*-*A* and contain reduced posterior body morphologies. **b**–**d.** Illustrations of arthropod taxa with reduced posterior segmentation and reported missing *abdominal*-*A*. **b** *Sacculina carcini* naupli (left; ventral view, oriented anterior up) and cypris (*right*; lateral view, oriented anterior up) (adapted from [107]). **c** *Tetranychus urticae*; lateral view, oriented anterior left. **d** *Endeis spinosa*; dorsal view, oriented anterior left (adapted from [108]). *Scale bar* in B = 5 cm, C = 0.125 mm, and D = 1 mm

In several arthropods there is a correlation between a reduced number of posterior segments and an absence of the posterior class Hox gene *abd*-*A*. *abd*-*A* was not identified in PCR surveys of Hox genes in three cirripede crustaceans (*Sacculina carcini*, *Elminius modestus*, and *Trypetesa lampas*) [34–36] and two chelicerates, including the pycnogonid *Endeis spinosa* [33] and the oribatid mite *Archegozetes* [11] (Fig. 3). The addition of *Tetranychus* to this list suggests that this correlation has emerged independently four times during arthropod evolution, once, in the lineage leading to the cirripedes, and three times within Chelicerata (Fig. 3). Although both *Tetranychus* and the oribatid mite are members of the acariformes lineage of chelicerates, the oribatid mites group more closely with the eriophyids, a group of mites that possess two pairs of legs and an elongated opisthosoma comprised of an uneven number of dorsal and ventral cuticular annuli that are not regarded as segments [90–92]. Based on the opisthosomal morphology of eriophyids, it might be expected that *abd*-*A* is present within this taxa and that loss of *abd*-*A* has occurred independently at least three times in the Chelicerata. However, a PCR survey of Hox genes and a molecular analysis of posterior body segments in eriophyids with a segmental marker such as *engrailed* have yet to be completed.

What role might the loss of *abd*-*A* have in the evolution of trunk morphology? It is possible that the loss of *abd*-*A* has no role in reducing the number of posterior segments and is merely a function of relaxed selection due to overlapping function with other Hox genes, e.g., *Ubx* and *Abd*-*B*. This may be similar to the loss, or derivation, of *Hox3* in insects [93]. Alternatively, the loss of *abd*-*A* could be a key evolutionary event underlying the reduction in the number of posterior segments. In vertebrate somitogenesis, which is superficially similar to arthropod sequential segmentation, termination of segmentation is promoted by the onset of expression of the vertebrate *Abdominal*-*B* homologs *Hoxb13* and *Hoxc13* [94, 95]. This late onset of expression is entwined with the temporal collinearity of vertebrate Hox genes, in which dynamic shifts in the three-dimensional chromatin arrangements within the Hox cluster occur during anteroposterior patterning [96, 97]. Again, while it is not well known whether arthropod Hox genes exhibit similar temporal controls overall, *Abd*-*B* is known to have a late onset of expression during segmentation in the apterygote *Thermobiadomestica* and the orthopteran *Schistocercagregaria* [77, 78]. In addition, during pupation *DrosophilaAbd*-*B* contributes to a reduction of posterior segments in a sex-specific manner [98, 99]. Taken together these data suggest that in arthropods that display reduced posterior segmentation, the loss of *abd*-*A* may have contributed to a change in the temporal chromatin dynamics of Hox clusters, transcriptional regulation of *Abd*-*B*, and an early termination of segmentation.

## Conclusions

The current data, from both genomic studies and PCR surveys, remain consistent with the idea that an ancestral arthropod had ten Hox genes. However, the accumulating non-insect Hox data raises questions as to whether ancestrally multiple Hox clusters were present and subsequently lost, and to what degree the ancestral arthropod clusters were dispersed within the genome. Vertebrate Hox genes are tightly clustered on the same chromosome with virtually no non-Hox genes interspersed within the cluster. Arthropod Hox genes are located on the same chromosome and in the same order and transcriptional orientation as their vertebrate orthologs, but they show varying degrees of dispersion. The Hox clusters in the genomes of *Daphnia* and *Strigamia* span relatively small genomic regions, while all chelicerates surveyed to date show a significantly more dispersed Hox cluster configuration, with the extreme example of the “atomized” cluster of the predatory mite. Within the insects surveyed here, there is an evident trend toward a greater dispersion of the Hox cluster within more derived species. Taken together, these data suggest the constraints maintaining Hox genes in a genomic cluster in arthropods have been relaxed in comparison with vertebrates and may play a functional role in the reduction of posterior body plans.

## Additional files

10.1186/s13227-016-0048-4 Gene accession numbers or genomic locations of Hox genes used in this analysis.
10.1186/s13227-016-0048-4  Positive BLAST hits and genomic locations of conserved Hox gene-associated miRNAs in *Mesobuthus*, *Metaseiulus*, *Tetranychus*, and *Strigamia.*

10.1186/s13227-016-0048-4  Number of BLAST hits, alignments, and sequences considered for conserved Hox gene-associated miRNAs in *Mesobuthus*, *Metaseiulus*, *Tetranychus*, and *Strigamia.*

10.1186/s13227-016-0048-4  Maximum likelihood phylogenetic tree of arthropod Hox gene-associated miRNAs. Abbreviations used: *Dm* – *Drosophila melanogaster*, *Ag* – *Anopheles gambiae*, *Tc* – *Tribolium castaneum*, *Nv* – *Nasonia vitripennis*, *Ap* – *Apis mellifera*, *Bm* – *Bombyx mori,* *Dp* – *Daphnia pulex*, *Sm* – *Strigamia maritima*, *Mm* – *Mesobuthus martensii*, *Is* – *Ixodes scapularis*, *Mo* – *Metaseiulus occidentalis*, and *Tu* – *Tetranychus urticae.*

10.1186/s13227-016-0048-4 Muscle alignment of arthropod miRNAs *mir*-*993*, *mir*-*10*, and *mir*-*iab*-*4/8*. Abbreviations used as in Additional file 4: Figure S1.
10.1186/s13227-016-0048-4  Calculated spatial relationship of Hox genes.
10.1186/s13227-016-0048-4 Global alignment of *Tetranychus fushi tarazu* and *Antennnapedia* nucleotide and protein sequences.
10.1186/s13227-016-0048-4 Maximum likelihood phylogenetic tree of *Antp* (A) and *ftz* (B). Phylogenetic trees were constructed with PhyML [64] using full length protein sequences aligned with MUSCLE. PhyML parameters were set as the following: WAG amino acid substitution model, proportion of invariable sites estimated, and the number of categories of substitution rate = 4. Statistical support was provided by approximate likelihood ratio tests based on a Shimodaira-Hasegawa-like procedure, with the scores shown in the tree.
10.1186/s13227-016-0048-4  RNASeq expression data for *Tetranychus urticae Antennapedia* and *fushi tarazu*. Data taken from embryonic (embryo_techrep_#), larval (larvae_techrep_#), nymphal (nymph_techrep_#), and adult (adult_techrep_#) developmental stages, values are reads per kilobase per million mapped reads (RPKM).
10.1186/s13227-016-0048-4 Measurements of the size of Hox gene coding and non-coding regions.

## Abbreviations

*lab*: *labial*
*pb*: *proboscipedia*
*zen*: *zerknüllt*
*Dfd*: *Deformed*
*Scr*: *Sex combs reduced*
*ftz*: *fushi tarazu*
*Antp*: *Antennapedia*
*Ubx*: *Ultrabithorax*
*abd*-*A*: *abdominal*-*A*
*Abd*-*B*: *Abdominal*-*B*
*miR*, miRNA: microRNA
*iab*: infra-abdominal
PCR: polymerase chain reaction
BLAST: basic local alignment search tool
MFE: minimum free energy
bp: base pairs
kb: kilobase pairs
Mb: megabase pairs
Gb: gigabase pairs
CDS: coding sequence
UTR: untranslated region
*M. musculus*: *Mus musculus*
*D. melanogaster*, Dm: *Drosophila melanogaster*
*A. gambiae*, Ag: *Anopheles gambiae*
*T. castaneum*, Tc: *Tribolium castaneum*
*N. vitripennis*, Nv: *Nasonia vitripennis*
*A. mellifera*, Ap: *Apis mellifera*
*B. mori*, Bm: *Bombyx mori*
*D. pulex*, Dp: *Daphnia pulex*
*S. maritima,* Sm: *Strigamia maritima*
*M. martensii*, Mm: *Mesobuthus martensii*
*I. scapularis,* Is: *Ixodes scapularis*
*M. occidentalis*, Mo: *Metaseiulus occidentalis*
*T. urticae*, Tu: *Tetranychus urticae*
