# Supplementary figures and images for: Composition and genomic organization of arthropod Hox clusters
Source: EvoDevo. 2016 May 10;7:11. doi: 10.1186/s13227-016-0048-4 (PMC4862073; doi:10.1186/s13227-016-0048-4)

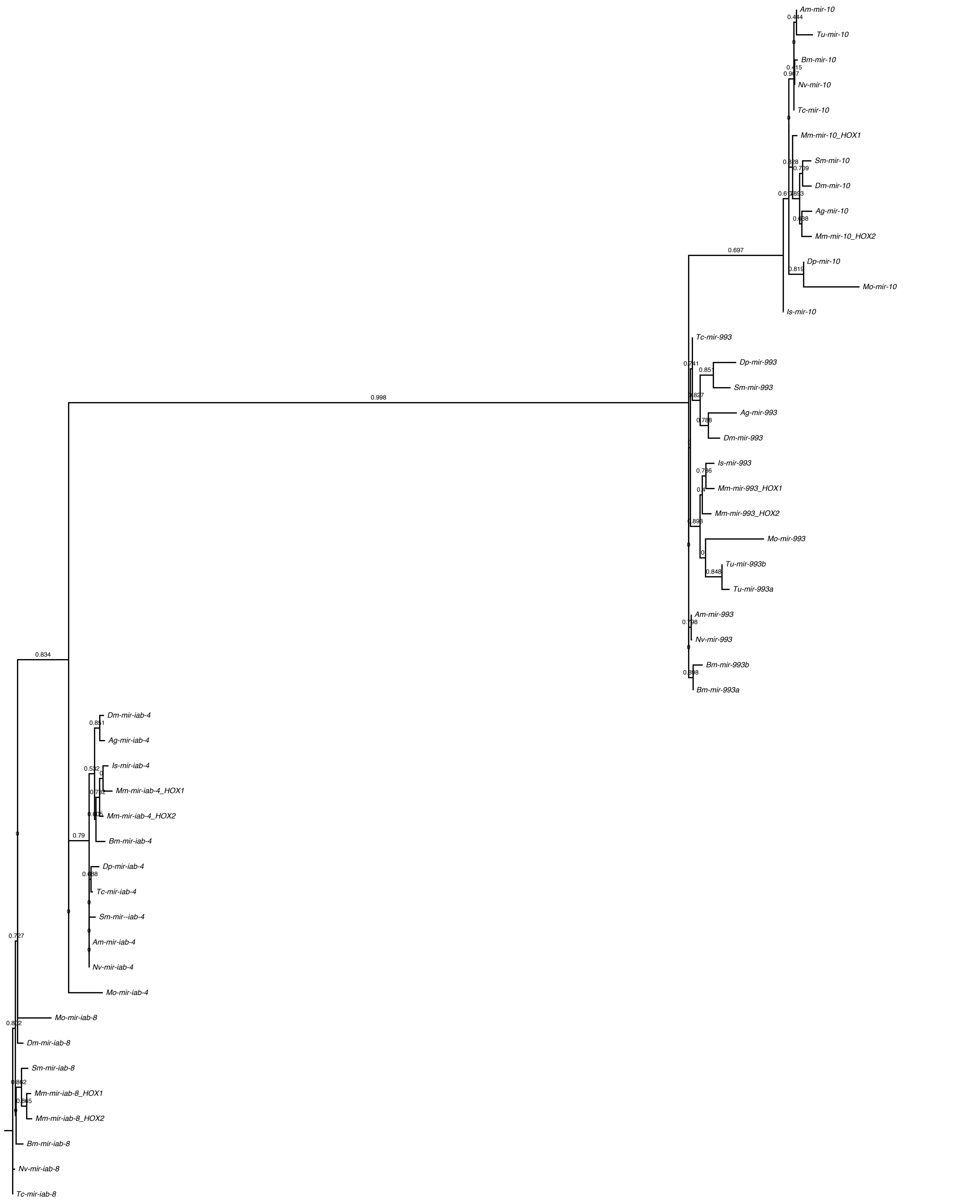

Supplement: Supplementary file 4 — 10.1186/s13227-016-0048-4 Maximum likelihood phylogenetic tree of arthropod Hox gene-associated miRNAs. Abbreviations used: Dm – Drosophila melanogaster, Ag – Anopheles gambiae, Tc – Tribolium castaneum, Nv – Nasonia vitripennis, Ap – Apis mellifera, Bm – Bombyx mori, Dp – Daphnia pulex, Sm – Strigamia maritima, Mm – Mesobuthus martensii, Is – Ixodes scapularis, Mo – Metaseiulus occidentalis, and Tu – Tetranychus urticae. [file 13227_2016_48_MOESM4_ESM.pdf]

A

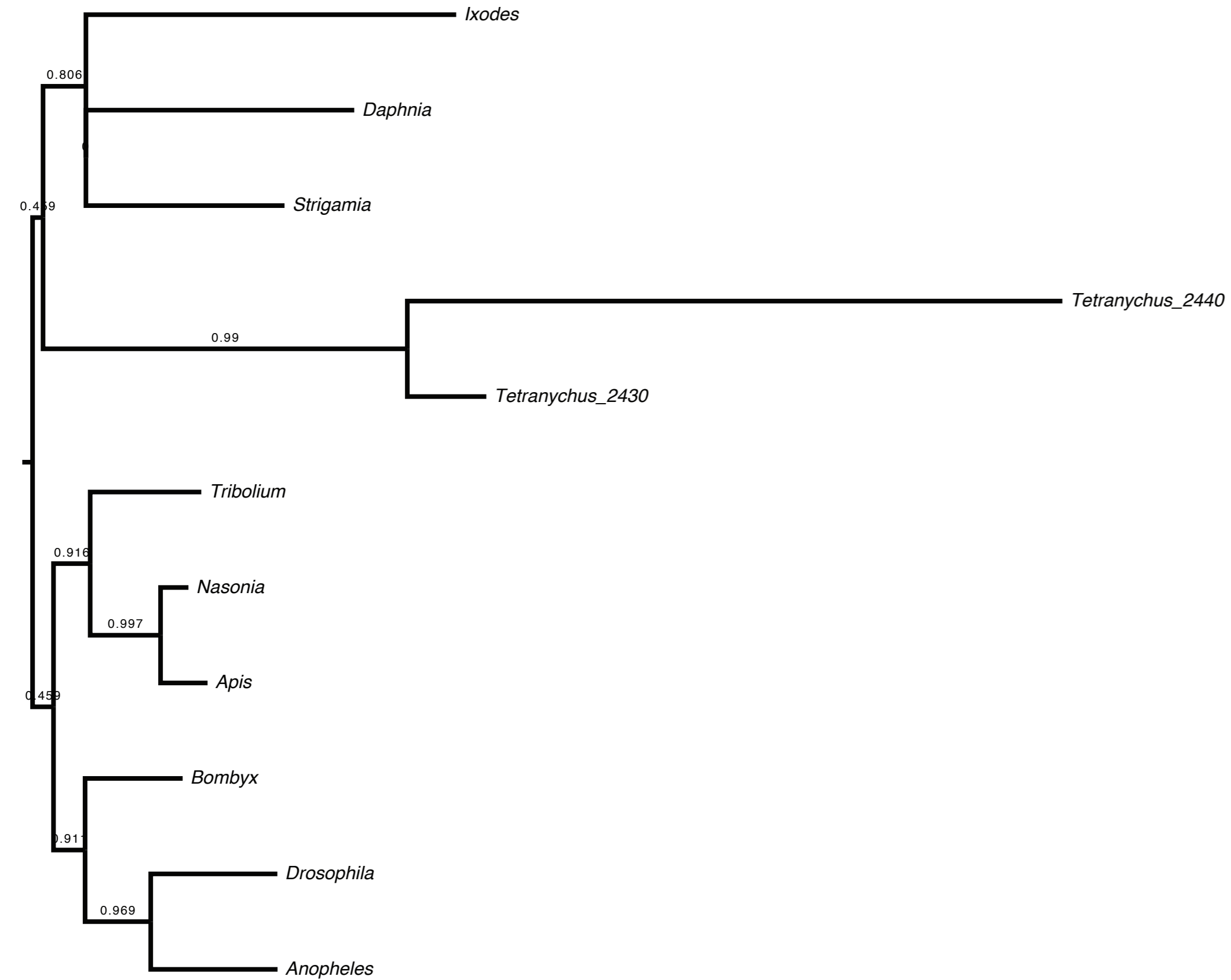

B

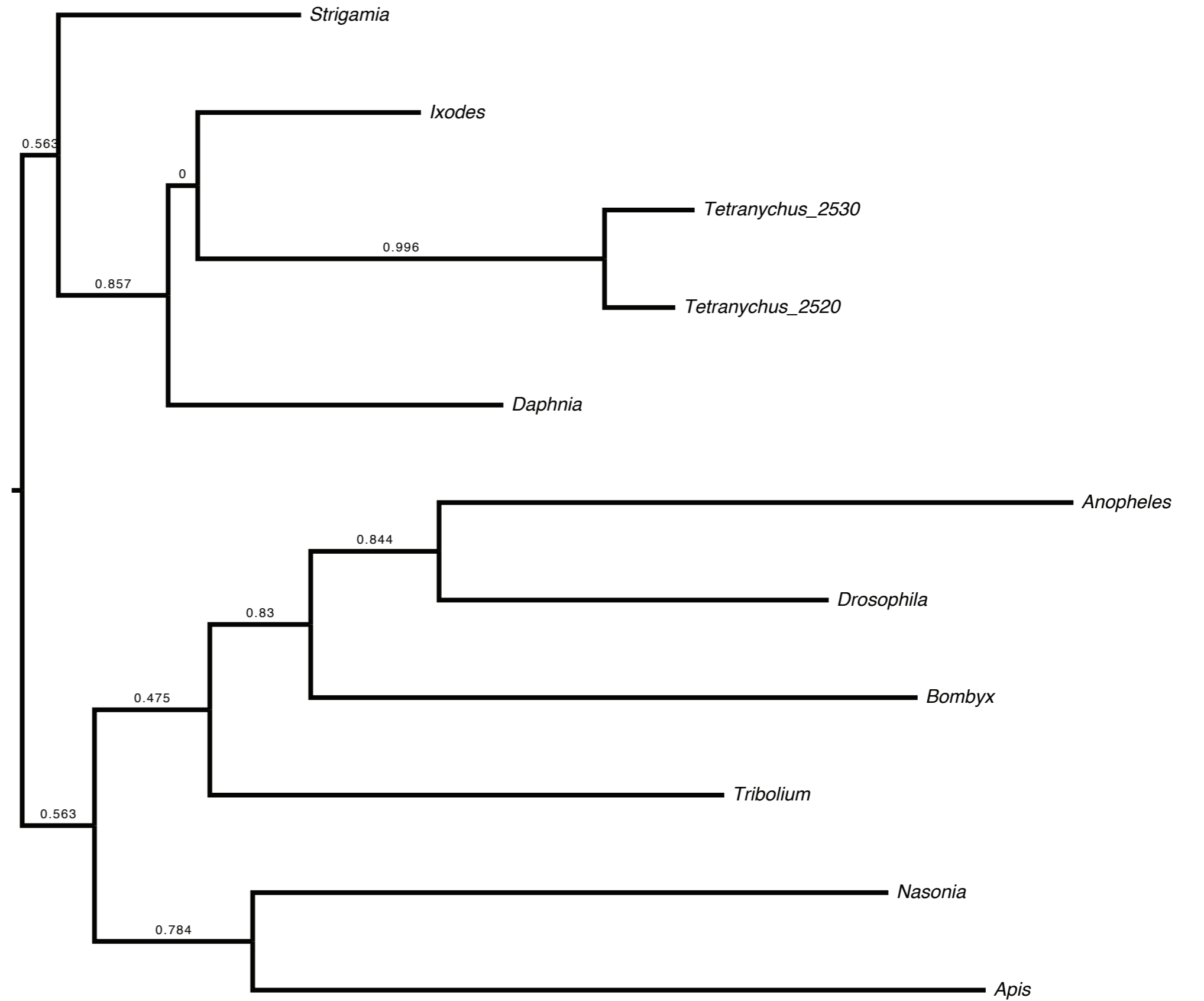

Supplement: Supplementary file 8 — 10.1186/s13227-016-0048-4 Maximum likelihood phylogenetic tree of Antp (A) and ftz (B). Phylogenetic trees were constructed with PhyML [64] using full length protein sequences aligned with MUSCLE. PhyML parameters were set as the following: WAG amino acid substitution model, proportion of invariable sites estimated, and the number of categories of substitution rate = 4. Statistical support was provided by approximate likelihood ratio tests based on a Shimodaira-Hasegawa-like procedure, with the scores shown in the tree. [file 13227_2016_48_MOESM8_ESM.pdf]
